# Effect of including fitness testing in preventive health checks on cardiorespiratory fitness and motivation: study protocol of a randomized controlled trial

**DOI:** 10.1186/1471-2458-14-1057

**Published:** 2014-10-10

**Authors:** Kirsten Høj, Mette Vinther Skriver, Anne-Louise Smidt Hansen, Bo Christensen, Helle Terkildsen Maindal, Annelli Sandbæk

**Affiliations:** Section of General Practice, Department of Public Health, Aarhus University, Bartholins Allé 2, Aarhus, C 8000 Denmark; Section of Health Promotion and Health Services, Department of Public Health, Aarhus University, Aarhus, Denmark

**Keywords:** Health examination, Cardiorespiratory fitness test, Exercise test, Cardiorespiratory fitness, Maximum oxygen uptake, Physical activity, Health behaviour, Motivation, Intention, Self-rated health

## Abstract

**Background:**

Preventive health checks may identify individuals with an unhealthy lifestyle and motivate them to change behaviour. However, knowledge about the impact of the different components included in preventive health checks is deficient. The aim of this trial is to evaluate whether including cardiorespiratory fitness testing in preventive health checks 1) increases cardiorespiratory fitness level and motivation to change physical activity behaviour and 2) reduces physical inactivity prevalence and improves self-rated health compared with preventive health checks without fitness testing.

**Methods/Design:**

An open-label, household-cluster, randomized controlled trial with a two-group parallel design is used. The trial is embedded in a population-based health promotion program, “*Check your Health Preventive Program*”, in which all 30–49 year-old citizens in a Danish municipality are offered a preventive health check. In each arm of the trial, 750 citizens will be recruited (1,500 in total). The primary outcome is cardiorespiratory fitness level assessed by submaximal cycle ergometer testing after one year. An intermediate outcome is the percentage of participants increasing motivation for physical activity behaviour change between baseline and two-weeks follow-up assessed using the Transtheoretical Model´s stages of change. Secondary outcomes include changes from baseline to one-year follow-up in physical inactivity prevalence measured by a modified version of the questions developed by Saltin and Grimby, and in self-rated health measures using the Short-Form 12, Health Survey, version 2.

**Discussion:**

This trial will contribute to a critical appraisal of the value of fitness testing as part of preventive health checks. The conduction in real-life community and general practice structures makes the trial findings applicable and transferable to other municipalities providing support to decision-makers in the development of approaches to increase levels of physical activity and improve health.

**Trial registration:**

ClinicalTrials.gov Identifier: NCT02224248. Registered 8 August 2014.

## Background

Changing an unhealthy lifestyle improves health and reduces morbidity [1]. Motivation is a key component in initiation and maintenance of lifestyle changes [2]. Identifying and motivating individuals with an unhealthy lifestyle to change behaviour may be achieved through preventive health checks as already implemented in some countries [3–5]. However, knowledge about the impact of individual components included in preventive health checks is deficient. Consequently, the most effective composition of preventive health checks remains unknown.

In a Danish municipality, the *Check your health preventive program* was recently initiated, offering preventive health checks to all 30 to 49 year-old citizens [4, 6]. This program provides the unique opportunity to evaluate single components of a preventive health check in a real-life setting. Only few previous studies have included cardiorespiratory fitness (fitness) testing in a preventive health check [7, 8]. Fitness level is associated with multiple health benefits such as improved cardio-metabolic profile and reduced risk of cancer, diabetes, and depression [9]. The primary determinant of fitness is physical activity behaviour [10]. Performing a fitness test may raise individual awareness of one´s actual fitness and lead to physical activity behaviour changes. The conception that measurement itself may influence behaviour is well-established and has already been applied in some physical activity promotion trials [11]. “Measurement reactivity” and “mere measurement” effect are some of the terms used to describe the phenomenon [12]. Increasing awareness and motivation have been proposed as explanations for the effect [13, 14]. A Danish report documented health status as being the most important motivational factor among physically inactive adults [15]. Conjoined, the realization of poor fitness and unfavourable health status may operate synergistic in motivating physically inactive individuals to change physical activity behaviour. Investigating the effect of including fitness testing in a preventive health check is therefore intriguing.

Our aim is to investigate the effect of including fitness testing in preventive health checks on 1) fitness level and motivation for changing physical activity behaviour and 2) changes in physical inactivity prevalence and self-rated health. We hypothesize that fitness testing as part of preventive health checks compared to preventive health checks without fitness testing 1) increases fitness level assessed after one year and the percentage of participants increasing motivation to change physical activity behaviour assessed after two weeks, and 2) reduces physical inactivity prevalence and improves self-rated health scores during the one-year study period. The study protocol conforms to the CONSORT Statement for randomized trials of non-pharmacological treatment.

## Methods

### Trial design

The trial is as an open-label, household-cluster randomized controlled trial with a two-group parallel design. The trial is nested in the ongoing health promotion program entitled *Check your Health Preventive Program* (CHPP) [4, 6]. The CHPP is carried out during the five-year period 2012 to 2017. The present trial will be conducted in 2014 to 2016, including a follow-up period of one year.

### Participants and settings

**Figure 1:**
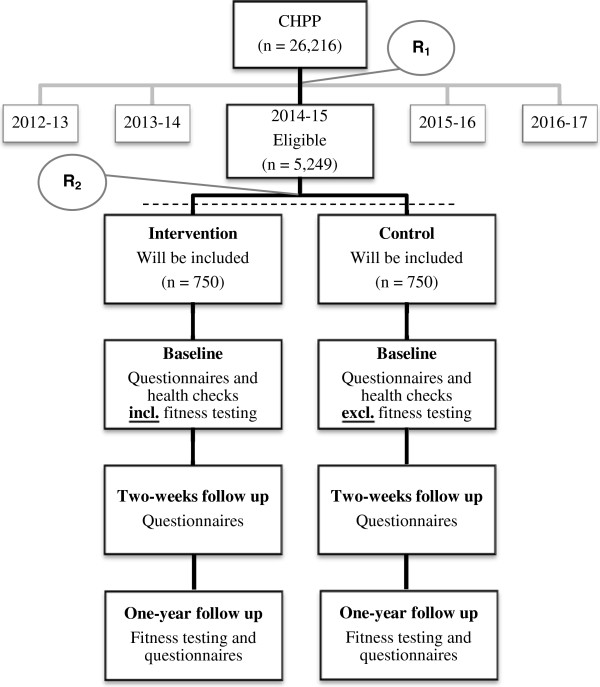
**Flow of participants in the CHPP and the present trial.** R_1_: Randomization by household to one of the five years in the CHPP. R_2_: Randomization by household to intervention or control in the present trial prior to dispatching the invitations. Invitation and inclusion proceed continuously until 750 participants in each arm of the trial have received a health check and consented for data to be used scientifically. Abbreviations: CHPP: Check your Health Preventive Program.

In the CHPP, all citizens living in the municipality of Randers aged 30–49 years per 1^st^ of January 2012 (n =26,216) were identified in the Danish Civil Register, in which a permanent and unique identification number is assigned to every Danish inhabitant at birth and to residents on immigration. The identified population was randomized into five equal groups, one for each year of the CHPP [6]. Citizens randomized for group three (n =5,249) are considered eligible for the present trial (Figure 1). A single exclusion criterion for not receiving an invitation in the CHPP is terminal illness as reported by the general practitioners (GPs) [6]. Invitations are dispatched continuously by mail and include information about the CHPP and a prefixed appointment for a health check. This appointment can be accepted, altered or rejected via phone or internet. If no response, a reminder with a new appointment is sent after 7 days and again after three weeks [6]. Randomization in the present trial is conducted prior to dispatching the invitations. The enrolment proceeds continuously, until 750 participants in each arm of the trial have received a health check and consented for data to be used scientifically. Health checks and health behavioural courses will take place at Randers Health Centre (RHC) and health consultations at the citizen´s GP.

### Intervention group

As part of the CHPP, the intervention group will answer a questionnaire and receive a preventive health check with fitness testing (Figure 1). Subsequent follow-up actions will proceed according to risk profile and individual choices (see Follow-up by risk).

### Questionnaire

Prior to the health check, a web-based questionnaire regarding physical activity level [16], motivation for changing physical activity behaviour [17], self-rated health [18], smoking habits [19] and alcohol risk behaviour [20] will be answered. Participants have the opportunity to answer the questionnaire at the RHC, if support is needed.

### Fitness testing

Fitness level will be assessed using a modified version of the Astrand submaximal cycle ergometer test [21] (Monark 939 E Pendulum Ergometer, Monark Exercise AB, Sweden) with a workload of 75 watt for women and 100 watt for men. Pedalling frequency is set between 60–70 rounds per minute. Heart rate will be measured continuously (polar T31 coded transmitter, Polar, Denmark) and recorded at 5.5 minutes, if participants achieve a steady-state pulse within a target interval of 120–170 beats/min. Steady state is defined as two consecutive heart rate recordings within 5 beats of each other [22]. If the target interval is not reached after 2 minutes of cycling, the workload will be increased with 35 and 50 watt for women and men, respectively. The test will proceed until a steady-state is reached. Fitness level will be estimated from the recorded heart rate and workload standardized to age and sex [21] (Monark 939 E Analysis Software, Version 3.0.9, Monark Exercise AB, Sweden). Blood pressure above 180/110 mmHg is considered a contraindication for performing the test. Moreover, the use of pacemaker or beta-antagonists results in exclusion from fitness testing due to unreliable heart rate responses [23].

### The health check

In addition to fitness testing, the health check will include assessment of body weight and height, waist circumference, systolic and diastolic blood pressure, biochemical measures (lipid profile, glycated haemoglobin (HbA1c)), and lung function. Moreover, cardiovascular disease a risk score is calculated. The health check, including all measurements, is described in detail elsewhere [6].

### Follow-up by risk

At the end of the health check, the results are presented in a personalized health profile leaflet, which includes recommendations for follow-up actions according to the risk-profile. Participants can either be referred to (a) a health-promoting consultation in general practice, (b) targeted health behavioural programs at RHC, or (c) no further follow-up. Participants advised to consult their GP are those with abnormal values of blood pressure, biochemical measures, lung function, self-rated health, including mental health, and alcohol risk behaviour [6]. Participants offered referral to behavioural programs are those with high body mass index and waist circumference, smokers, and physically inactive individuals. Participants referred to general practice can be referred to these programs by their GP. Participants with no risk factors receive no further follow-up. The stratification-algorithm and the behavioural programs are previously described [6].

### Control group

As part of the CHPP, the control group will answer the same questionnaire as the intervention group and receive an identical preventive health check *without* fitness testing (Figure 1). Subsequent follow-up procedures are equal to those described for the intervention group.

### Standardization and education of health care professionals

The health check will be conducted by health professionals trained in all measurement procedures and risk communication to ensure standardization and quality [6]. Execution of the health check is further standardized by a written protocol, to which adherence is checked continuously. As a part of the CHPP, the GPs received education in physical activity promotion and shared decision-making [6]. There is no standardization of the GPs handling of the results from the health check.

### Randomization and implementation

Randomization is handled by a data-manager with no scientific involvement. The eligible population for this trial was defined by the CHPP randomization, which was performed on clusters defined by households based on addresses obtained from the Danish Civil Register [6]. Using the same index individual per household, citizens randomized to group three in the CHPP will be further randomized by household to either intervention or control in the present trial (Figure 1). In practice, every second week will be scheduled for intervention group participants and the remaining weeks for control group participants. Group assignment will be maintained regardless of changes in the initially assigned appointment. Implementation and management of the booking-system is handled by a data-manager. The intervention, the outcomes, group assignment, and the future outcome assessments in this trial are unrevealed in the invitation for the CHPP. The health behavioural courses and the health consultations will be carried out unblinded due to the real-life setting. At the one-year follow-up, the personnel assessing the outcomes will be independent and blinded to group allocation.

### Outcome assessments

The intervention group and the control group will receive identical outcome assessments at two evaluation contacts. All participants will receive a questionnaire regarding motivation for changing physical activity behaviour two weeks after the health check and will be invited for fitness testing and asked to fill out a questionnaire after one year (Figure 1).

### Primary outcome

#### Fitness level

The criterion measure of fitness is maximal oxygen uptake, which can be expressed in relative (mL O_2_/min/kg) or absolute (L O_2_/min) terms [23]. Fitness level will be assessed as both a continuous and a categorical outcome. Specifically, mean levels of absolute and relative fitness will be assessed, and participants will be categorized according to their relative fitness level into five fitness groups (low, fair, average, good, high) based on pre-specified cut-offs [21]. The prevalence of low fitness is the specific categorical outcome of interest.

### Intermediate outcome

#### Motivation for physical activity behaviour change

Motivation for physical activity behaviour change will be operationalized by conceiving *intention* (to adopt a specific behavioural criterion) as a motivational construct. The Transtheoretical Model will compose the theoretical framework using the stages of change [17]. Stages of change conceptualize behaviour change as a progression through a series of five stages: *precontemplation* (*no intention* to engage in regular physical activity within the next six months), *contemplation* (*intention* to engage in regular physical activity *within the next six month*), *preparation (intention* to engage in regular physical activity *within the next 30 days), action* (regular physical activity behaviour initiated), and *maintenance* (regular physical activity has been performed for 6 month or more) [17, 24]. The three initial stages represent the motivational phase, in which intention is thought to increase [17]. We will consider progression from one of these three stages at baseline to a higher stage assessed two weeks after the health check as a motivational increase [17] and the percentage of participants increasing motivation as the specific outcome. Participants will stage themselves into one of the five stages according to their intention to engage in regular moderate physical activity defined as occurring on 5 or more days a week for at least 30 minutes (or 3 × 10 minutes) [23]. Moderate intensity is defined as producing increased heart rate, but being able to converse.

### Secondary outcomes

#### Physical inactivity

Leisure time physical activity level will be assessed using modified questions originally developed by Saltin & Grimby [16]. Participants will be asked to categorize their typical leisure time physical activity level as: ‘1. Mainly sedentary’ (e.g. reading, watching television or movies); ‘2. Low physical activity level’: engaging in light physical activities for more than four hours per week (e.g. leisurely walking, leisurely cycling, light do-it-yourself tasks, light house chores, table tennis, and bowling); ‘3. Moderate physical activity level’: engaging in sports or exercises minimum three times per week or vigorous leisure time activities (e.g. heavy gardening); or, ‘4. High physical activity level’: engaging in competitive sports or long distance running several times per week. Physical inactivity is defined as the lowest of the four activity categories (category 1). Change in reported physical inactivity prevalence from baseline to one-year follow-up composes the outcome.

### Self-rated health

Self-rated health will be assessed by the validated Danish version of the Short-Form-12 Health Survey, version 2 [18, 25]. It consists of 12 items covering 8 health domains of functioning and well-being, from which three self-rated health measures can be derived: a general health score, a physical component summary score, and a mental component summary score. All scores will be calculated by the standard scoring algorithm (U.S. derived) and presented as t-scores based on U.S. general population norms. The specific outcomes are changes in mean scores from baseline to one-year follow-up.

### Sample size

The sample size is estimated to be 1,500 participants in total. This sample size allows for a categorical analysis of fitness level and is calculated on the basis of the following assumptions: 1:1 randomization, false positive error rate of 0.05, power of 0.8, intracluster correlation coefficient of 0.05 and categorical analysis with a power to detect a difference of 10% in the prevalence of very low fitness between the study groups. We determined this 10% difference to be clinically meaningful based on expert opinions and criteria employed in other research [26]. The intracluster correlation coefficient was included to reflect a possible clustering effect of the GPs (n =46), which is seldom greater than 0.05 in primary care settings [27]. The estimated sample size accounts for a 30% loss to follow-up.

### Statistical methods

Statistical analysis will be performed using STATA 12.0 software. Continuous variables are presented as mean ± standard deviation and categorical variables as absolute numbers and relative (%) frequencies. In the comparative analyses Student´s t-test will be used, when comparing means or changes in means of continuous variables and Chi^2^-test or Fisher’s exact t-test, when comparing proportions for categorical variables. Analyses will be adjusted for baseline physical activity. Stratified analyses will be performed on sex and age groups and analysis of motivation will be performed on a subgroup comprising precontemplaters, contemplaters, and preparators at baseline. All analyses will follow the intention-to-treat principle [28]. If appropriate, multiple imputation methods will be applied [29] (using data from social and medical national registries coupled with health check data), and sensitivity analyses will be performed. Moreover, the potential effect of clustering by the GPs will be investigated. The statistical significance level is set at p <0.05.

### Ethics and legal aspects

The trial was presented for The Central Denmark Region Committees on Health Research Ethics and approval was not found necessary. The trial will comply with The Declaration of Helsinki and each participant will provide written informed consent for data to be used for research purposes in agreement with the Danish Health Law. Approval by The Danish Data Protection Agency is obtained (2013-41-2527) and the trial is registered at ClinicalTrials.gov [Identifier: NCT02224248].

## Discussion

Various diagnostic tests have been applied in preventive health checks ranging from simple anthropometrical measures and blood pressure to broad biochemistry panels and x-rays [30]. However, due to the multiple components often included in preventive health checks, the effect of individual components cannot be apportioned. Accordingly, decision-makers contemplating implementation of population-wide preventive health checks have poor requisites to make evidence-based choices regarding the content of such health checks.

To the best of our knowledge, this randomized controlled trial is the first to investigate the effect of fitness testing as part of preventive health checks on fitness and motivation for changing physical activity behaviour in a population-based, real-life setting. Generally, the effect of mere measurement on actual physical activity behaviour has received very little attention [11]. A study by Sluijs et al. investigated the effect of physical activity measurements based on questionnaires and accelerometers [13]. This study found a positive effect of questionnaire-based measurements on participants’ awareness of meeting physical activity guidelines and on their level of physical activity after six months [13]. By contrast, no effect was demonstrated for the accelerometer-based measurements [13]. The authors explained this latter finding by the popularity of cycling in the Netherlands and the inability of hip-worn accelerometers to accurately pick up cycling [13]. Performing a fitness test may raise a bodily awareness of one´s actual fitness level that may not be attained by answering a questionnaire or wearing an accelerometer. This awareness may lead to a better understanding of the consequences of one´s physical activity behaviour.

### Evaluation choices

Every improvement of fitness is important for health and longevity [31–33]. However, the greatest gain results from improving fitness at the low end of the fitness-range [34]. On this basis, we chose to evaluate the effect of fitness testing on both mean level of fitness and the prevalence of very low fitness in the primary analysis. Longitudinal studies have shown that only 50% of participants follow-through on positive intentions to change physical activity behaviour [35]. Moreover, a majority of those succeeding may relapse to a less active or inactive status during the one-year follow-up period [36]. Thus, to acquire information about fitness testing as a motivational facilitator in preventive health checks, the stages of change was integrated as part of the evaluation. Adjacent stages have been successfully distinguished by intention to participate in strenuous, moderate, and mild exercise for adults [17, 37]. Engaging in regular moderate physical activity is shown to be sufficient to provide fitness improvements [38]. Hence, from the view that becoming moderately physical active is more achievable than becoming strenuously physical active, we chose a definition of regular moderate physical activity to stage participants. However, significant differences have been found only among the early stages and not between action and maintenance [17]. The analysis of this outcome will therefore be performed only on a subgroup.

In the secondary analyses, change in the prevalence of physical inactivity was included due to the existing evidence of a beneficial health effect of physical activity, independently of fitness [39], especially among the least active [40]. To broaden the perspective on the value of fitness testing, changes in general-, physical-, and mental self-rated health scores was included as well. Self-rated health is a subjective construct reflecting the individual´s integrated perception of health, and an association with mortality is well-established [41]. There have been some disagreements regarding the method for constructing the physical- and mental summary scores [42]. However, similar results have been demonstrated across different scoring algorithms [42].

### Feasibility

Including fitness testing in population-based preventive health checks is subject to some feasibility considerations. While maximal fitness tests are considered more accurate than submaximal fitness tests, they require participants to exercise to the point of volitional fatigue, which is more demanding on participants, time-consuming and labour-intensive [23, 43]. The submaximal fitness test employed in the present trial is easier to perform, which ensures that most people are able to complete the test. It is more convenient in terms of time, effort and cost, yet still provides adequate estimates of fitness [10, 44].

### Strengths and limitations

The present trial holds several strengths. Participants will be recruited directly from the community receiving no screening prior to inclusion. Thereby, the generalizability will not be restricted in advance to certain at-risk populations such as physically inactive people or people with chronic diseases. Moreover, the risk of diluting an intervention effect by prerandomization research activities [12] is omitted. The objective measure of fitness provides effect estimates with high internal validity. Furthermore, the one-year follow-up period and the intention-to-treat approach form the basis of high external validity.

Some important limitations also deserve consideration. Firstly, although the Astrand method of predicting fitness has been shown valid at a group-level [44], the risk of misclassification exists at the individual level [43]. Fitness will be assessed identically in the two groups being compared. Hence, any misclassification would be non-differential, and the resulting bias would lead towards null. Secondly, contamination may occur across social groups and in the community. In recent years, the municipality of Randers has implemented health promotion initiatives, which may impact on trial outcomes leading to further attenuation of the intervention effect. Thirdly, blinding of health professionals and GPs is impossible in a pragmatic trial such as this. Consequently, the risk of performance bias cannot be ruled out. Finally, a full application of the intention-to-treat principle requires complete outcome data for all randomized subjects [45]. This is rarely possible in pragmatic trials, in which losses to follow-up are common and introduce a risk of selection bias [45]. Nevertheless, no consensus exists about how missing outcome data should be handled in intention-to-treat analyses [45]. We will address potential bias by imputing missing outcome data and performing sensitivity analyses.

### Perspectives

This trial will contribute to a critical appraisal of the value of fitness testing as part of preventive health checks. The conduction in real-life community and general practice structures makes the trial findings applicable and transferable to other municipalities providing support to decision-makers in the organization of population-wide preventive health checks. Overall, the trial may contribute to the development of approaches whereby an increase in physical activity at a community level can be achieved.

## Abbreviations

CHPP: Check your health preventive program
Fitness: Cardiorespiratory fitness
GP: General practitioner
RHC: Randers health centre.
